# The Comprehensive Utilization of Bean Dregs in High-Fiber Tofu

**DOI:** 10.3390/foods11101475

**Published:** 2022-05-19

**Authors:** Wenjing Lu, Yue Zhang, Chaogeng Xiao, Di Chen, Qin Ye, Cen Zhang, Xianghe Meng, Shengjian Wang

**Affiliations:** 1 Institute of Food Science, Zhejiang Academy of Agricultural Sciences, 298 Desheng Road, Hangzhou 310021, China; wenjing_316@163.com (W.L.); cd0sindy@163.com (D.C.); yqin719@163.com (Q.Y.); cenzhang@zju.edu.cn (C.Z.); 2 College of Food Science and Technology, Zhejiang University of Technology, Hangzhou 310014, China; 15957104218@163.com (Y.Z.); mengxh@zjut.edu.cn (X.M.); 3 Hangzhou Bean Food Co., Ltd., No. 18, Fengdu Road, Fengdu Industrial Park, Hangzhou 311115, China; wangshengjian@163.com

**Keywords:** high-fiber tofu, bean dreg, GC-IMS, nuclear magnetic resonance

## Abstract

A large quantity of bean dregs is produced by the production of tofu and treated as animal feed or plant fertilizer, which could cause environmental pollution. The purpose of this study was to use commercially available lactone tofu to compare the effects of innovative preparation methods of high-fiber tofu, where the innovative methods used partial de-slagging followed by the addition of soybean residue cellulose to prepare high-fiber tofu. The results showed that there were no significant differences among lactone tofu samples made with 5% cellulose, 10% cellulose, or 15% cellulose and the commercially available lactone tofu during the water-holding capacity and chroma analysis. Texture indices showed that lactone tofu with 10% cellulose was similar to the commercially available lactone tofu in chewiness and hardness, and lactone tofu with 15% cellulose was similar to the commercially available lactone tofu in adhesiveness and chewiness. Magnetic resonance imaging displayed that lactone tofu with 10% cellulose had better water retention and higher moisture content. Gel electron microscopy showed that lactone tofu with 10% cellulose achieved a better gel network, and the bean dreg cellulose had less influence to a certain extent. Volatile organic compound testing by GC-IMS method indicated that the lactone tofu with 10% cellulose had more volatile organic compound content. In conclusion, these results demonstrated that lactone tofu with 10% cellulose had the best market competitiveness in ensuring the quality of high-fiber tofu.

## 1. Introduction

Soybeans are the main legume grain crop in China, with annual soybean production in China at 16.4 million tons in 2021 [1]. As the main waste produced by processing bean products such as tofu and soy milk, bean dregs have long been treated as animal feed or plant fertilizer because of high storage costs and sometimes even abandonment [2,3,4]. In the long run, it not only wastes resources but also causes environmental pollution.

Although bean dregs are rich in nutrients, and especially rich in high cellulose and protein, there are some problems with their use [5]. First, the unsaturated fatty acids in the bean dregs are prone to oxidation and produce a special flavor of soybean meal. Second, the bean dregs contain a lot of moisture in the summer, and they easily breed bacteria and deteriorate. Third, the dietary fiber content the bean dregs is high, but it is a difficult flavor in conventional eating and is difficult to market. If the dietary fiber in bean dregs can be efficiently extracted and recycled, it will not only improve the utilization rate of soybeans but also contribute to environmental protection [6].

Tofu is a popular Asian food that is made by coagulating soymilk and pressing the curds into soft white blocks [7,8]. Coagulants such as CaSO_4_, CaCl_2_, and gluconolactone (GDL) are used to coagulate soymilk [8]. This traditional method of processing tofu produces a large quantity of bean dregs, thereby reducing the yield of tofu and causing waste. Tofu is high in phenolic compounds such as isoflavones, which have antioxidant properties and health benefits, because it is made from soybeans [7,8,9,10]. Tofu includes essential nutritional components, but it lacks dietary fiber due to the removal of the soybean pulp during traditional manufacture [10]. Meanwhile, bean dregs are used mainly as animal feed [2]. As feeding patterns change, the consumption of bean dregs is shrinking [3]. Whole-bean high-fiber tofu would not only improve the nutritional value of tofu but also solve the problem of bean dreg waste. However, due to the presence of large fiber particles in the bean dregs formed during the production process, their direct addition to soybean milk weakens the gelation between the proteins, which may directly affect the form of the tofu gel [9,10,11,12]. Ultrasonic technology to assist the biological enzyme to treat the bean dregs reduces the particle size of the fiber, and the protein and lipid in the bean dregs can be removed to solve the problem. At present, there are few studies on the addition of bean dregs to soy milk to make tofu. In previous reports, the influence and research on the texture of the bean curd on the tofu were not comprehensive and in-depth, and the evaluation indices had no specific experimental process [10]. In some reports, micro-pulverized bean dreg fiber extract was added to the tofu, and the effects were investigated of the number of bean dregs on the gel properties of tofu, its water-holding color, and other evaluation indices through the combination of texture, evaluation, and comparative analysis experiments [13]. The effect of dietary fiber (crude fiber) in soybean residue on the soy protein-forming tofu gel system was discussed.

There have also been tremendous efforts to produce dietary fiber from vegetable sources as a functional ingredient. Baked goods fortified with dietary fiber, fiber-enriched cocoa powder, dietary fiber-enriched biscuits made with okra flour, and whole wheat/soy flour bread are only a few examples [14]. However, there is little research to validate the effects of adding dietary fiber to tofu, particularly utilizing soybean residual cellulose as a source of dietary fiber.

Consumer desire for unprocessed or minimally processed whole foods is growing, in addition to the health advantages of dietary fiber. Enriching tofu with dietary fiber from soybean residue could increase the level of dietary fiber in tofu and meet the rising consumer desire for complete meals [13]. This study mainly discusses the production of whole bean tofu in an innovative process using whole soybeans as raw materials; improving the nutritional value of the products, especially fiber content, could improve the utilization rate of soybeans and solve the problem of large-scale bean dreg waste. Moreover, based on the traditional manufacturing method, the innovative production method of high-cellulose tofu not only ensured the texture and taste but achieved the goal of recycling bean dregs and realized the comprehensive utilization of innovative processes.

## 2. Materials and Methods

### 2.1. Materials

Soybean were purchased from Heilongjiang Province, China. Gluconic acid lactone was purchased from Xin Huanghai food Co., Yibin, China. Trypsin, alkaline protease, and papain were purchased from Lingfeng reagent Distribution Department (ShangHai, China).

### 2.2. Determination of Nutrients in Bean Dregs

The soybean residue was analyzed for moisture, protein, and other chemical constituents. Moisture was determined using an oven method (AOAC International, 2012; Method 934.01). Crude protein was determined using the Kjeldahl method (AOAC International, 2012; Method 955.04). The nitrogen-to-protein conversion factor was 6.25. 

### 2.3. Analysis of Water-Holding Capacity

The centrifugation procedure was performed according to Urbonaite et al. (2014) [15]. A total of 2.0 g (m_0_) tofu sample was weighed to m_1_, and 30 mL of deionized water was added and mixed for 5 min. The mixture was centrifuged at 4500 rpm min^−1^ for 15 min, then remaining distilled water was drained off. The moisture attached to the inner and outer walls of the centrifuge tube were the total mass of the substance, which was m_2_. The water-holding capacity (g/g) is calculated according to the following formula:

Formula: Water-holding capacity = (m_2_ − m_1_)/m_0_ × 100%.

### 2.4. Extraction of Bean Dreg Cellulose

This method combined the ultrasonic-assisted enzymatic extraction of water-insoluble cellulose by Zhang and Pan [11,12]. The fresh bean dregs were dried at 80 °C for 6 h and milled with a high-speed grinder for 5 min, and the process was repeated three times until the bean dregs were ground into powder. A total of 10 g powder added with 100 mL distilled water was boiled for gelatinization of amylase. Then, 0.01 g, 0.02 g, 0.03 g, and 0.04 g alkaline protease; 0.05 g, 0.1 g, 0.15 g, and 0.2 g trypsin; and 0.004 g, 0.005 g, 0.006 g, and 0.007 papain were added with 10 g bean dreg powder to removing protein. To decompose the water-insoluble cellulose into water solubility and reduce cellulose particle size, 0.1 g, 0.15 g, 0.2 g, and 0.25 g cellulose were added to 10 g bean dreg powder.

### 2.5. Preparation of High-Fiber Tofu

Following the tofu production method of Cheng et al. (2004) [13], the soybeans were added to distilled water after washing repeated times, and then the soybeans were frozen at −4 °C for 12 h. Soy milk was made from the soybeans using a broken wall machine and shearing machine. The bean dregs were removed, and cellulose was extracted from the bean dregs, and then the cellulose was returned to the soy milk without the bean dregs. The soy milk with additional cellulose was ultrasonicated (40 KHz, 60 min, 55 °C) and heated to 100 °C. The slurry was added to glucose lactone when the slurry had cooled to 90 °C, and then the slurry was insulated at 90 °C for 35 min. At last, the condensate was put into the mold, and high-fiber tofu was produced after pressuring and removing water.

### 2.6. Texture Profile Analysis

The full texture profile assay aimed to simulate human chewing of food. The physical texture parameters were obtained by the force and time profiles obtained through the textured probe according to Zhang et al. (2013) [16]. The tofu from different production processes was stored at 4 °C for 12 h and returned to room temperature before measurement. The texture of the tofu was analyzed according to a texture profile analysis (TPA) using a texture analyzer (TA.XT.plus, Stable Micro Systems, Surrey, UK) with a 35 mm diameter compression plunger. Each tofu sample was cut into 1 cm cubes. Each cylindrical sample was placed on the center of the TPA plate and compressed twice to 45% of its original height by a cylinder probe (P 36R) at a constant speed of 1.0 mm/s. The texture profile analysis curve was recorded, and the hardness, adhesiveness, springiness, cohesiveness, and chewiness were calculated automatically [16]. 

### 2.7. Chroma Analysis

This method was slightly modified from Kim’s color measurement [14]. The color of tofu was determined based on CIE using a colorimeter (UltraScan Pro, HunterLab, Reston, VA, USA). Each sample was taken from three different parts and averaged. Before the sample was analyzed, the standard whiteboard was used to correct the color difference meter to determine the sample b* (yellowness value; a positive number represents yellow, and a negative number represents blue), a* value (redness value of reaction sample; a positive number represents red, a negative number represents green), L* (luminance value, brightness of reaction color).

### 2.8. Microstructure of Tofu

This method was modified from Ullah’s (2019) approach [17]. The prepared central portions of the tofu samples of different processes were cut into small squares of 2 mm × 2 mm × 2 mm and fixed in a 2.5% glutaraldehyde solution at 4 °C overnight, and the fixed samples were used in a fume hood. The sample was rinsed three times with 0.1 M phosphate buffer solution (pH 7.0) for 15 min each time, then the sample was fixed with 1% citric acid solution for 1–2 h, and the fixative solution was poured off. The samples were dehydrated with 50%, 70%, 80%, 90%, and 95% (*v*/*v*) ethanol solution, and each treatment was operated for 15 min at each concentration. The sample was treated twice with 100% ethanol for 20 min. The sample was then treated with a mixed solution of ethanol and isoamyl acetate (*v*/*v* = 1/1) for 30 min, and then treated with pure isoamyl acetate for 2 h; then the treated sample was dried at a critical point. The sample was fixed to the sample stage with conductive double-sided tape, and the surface morphology of the sample was observed under a scanning electron microscope after gold plating treatment [17]. The SEM micrographs of samples were recorded at 15 kV.

### 2.9. Volatile Compounds Determination

Instrumentation and software analyses of volatile compounds were performed on a FlavourSpec^®^ Static Headspace (SHS) (https://www.imspex.net/flavourspec/ (accessed on 8 April 2022)) Gas chromatography-ion mobility spectrometry (GC-IMS) instrument (Gesellschaft für Analytische Sensorsysteme mbH (G.A.S.), Dortmund, Germany) with a heated splitless injector. The ionization source of the IMS was tritium 3H. The device was equipped with an autosampler (PAL RSI, CTC Analytics AG, Zwingen, Switzerland) and fitted with a WAX-30 m × 0.53 mm ID capillary column (CS-Chromatographie Service GmbH, Düren, Germany). IMS instrument data were acquired in positive mode and evaluated using LAV^®^ software (G.A.S.) (https://www.gas-dortmund.de/Products/Software/Laboratory-Analytical-Viewer/1_463.html (accessed on 8 April 2022)) including Reporter, Gallery Plot and Dynamic PCA plugins. Moreover, GCxIMS Library Search^®^ software (G.A.S.) (https://www.gas-dortmund.de/index-gas.php?spath=464 (accessed on 8 April 2022)) was employed to identify compounds.

### 2.10. GC-IMS Method

The method used to analyze the volatile compounds in greengage wine was modified on several parameters [18,19,20,21]. The headspace (100 μL) was sampled and automatically injected using a heated syringe (85 °C). Carrier gas (N_2_ with an inlet pressure of 3 bar) was passed through the GC-IMS injector, introducing the sample into the capillary column. Analytes were isothermally eluted at 60 °C and driven to the ionization chamber. Compounds were ionized by the 3H source at atmospheric pressure, yielding product ions (protonated monomers or proton-bound dimers) that depended on the analytes’ concentration and chemical nature. Then, the ions were pushed into a 9.8 cm length drift tube through the shutter grid, which operated at a constant voltage (500 V cm^−1^) and temperature (45 °C). The drift gas (N_2_) flow was set at 150 mL min^−1^. Each spectrum had an average of 16 scans with a repetition rate of 30 ms. The double separation obtained in the GC column and the IMS drift tube are represented in a topographic plot. Each feature in this plot is defined by a retention time, drift time, and intensity value.

### 2.11. Analysis and Detection of the Magnetic Resonance Imaging System

Nuclear magnetic resonance (CPMG) sequence relaxation measurements were performed on a Newman benchtop pulse NMR analyzer PQOO1 [22]. The proton resonance frequency was 21 MHZ, and the measurement temperature was 25.7 °C. Approximately 2 g of the sample was placed in a 15 mm diameter nucleus tube and placed in the analyzer for testing. The parameters used for the transverse relaxation time T_2_ measurement were as follows: the t-value (90°–180° pulse interval) was 150 μs; the scan was repeated 16 times; the interval between adjacent repeated measurements was 3 s; and the number of echoes was 3000 [22]. There were two replicates per sample. MultiExp software was used for the inversion of nuclear magnetic data. The continuous multi-exponential distribution of the CPMG experiment could be defined by the following equation:

(1) $$R_{mag}\left(t\right)=\overset{n}{\underset{j=1}{\sum }}P_{2j}\;exp\left(\frac{-t}{T_{2j}}\right)+L$$

where *R_mag_* was the peak of the echo that decays at time *t*. *P*_2*j*_ and *T*_2*j*_ are the intensity of the relaxation component j and the corresponding relaxation time, respectively. *L* represents curve noise. The software uses the Laplace transform inversion algorithm, and the result is a combination of discrete and continuous patterns. Each peak time constant *T*_2*i*_ (peak time) and its area fraction *M*_2*i*_ were recorded for subsequent analysis.

T_2_ samples are CPMG sequences:

Inherent parameters: SF = 21 MHz, O_1_ = 238,762 Hz, P_1_ = 5.4 μs, P_2_ = 10.8 μs.

Adjust parameters: SW = 250 KHz, TW = 6000 ms, RFD = 0.15 ms, RG1 = 20, DRG1 = 3, NS = 4, DR = 1, PRG = 1, NECH = 18,000, TE= 0.8 ms

### 2.12. Data Analysis

One-way ANOVA was performed using SPSS 17.0 statistical software, and multiple comparisons were performed using the LSD method (*p* < 0.05, significant difference), and the same letter indicates that the difference between data is not significant (*p* > 0.05), and different letters represent significant differences (*p* < 0.05). Data were expressed as mean ± standard deviation (SD) of at least three tests.

## 3. Results and Discussion

### 3.1. Analysis of the Content of Nutrients in Bean Dregs

The main nutrients in bean dregs were protein and crude fiber, and the contents of ash and fat were low, proving that the highest content of soybean residue is water (Table 1). Therefore, removing the protein and crude fiber was the primary step in the extraction of bean dregs. This result was consistent with a previous study on the nutritional composition of soybean residues by Li et al. (2012) [23].

### 3.2. Extraction of Bean Dregs Cellulose

**Figure 1:**
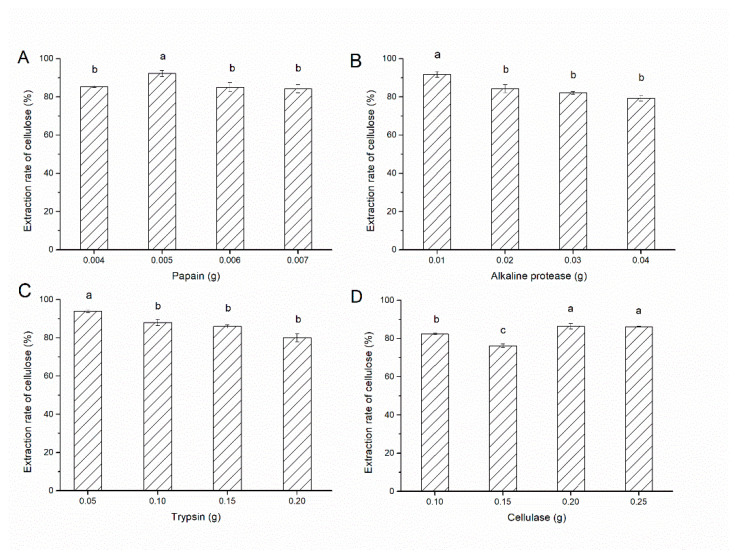
The effects of adding different proteases on the extraction of cellulose. (**A**) The effect of papain addition on the extraction of cellulose. (**B**) The effect of alkaline protease addition on the extraction of cellulose. (**C**) The effect of trypsin addition on the extraction of cellulose. (**D**) The effect of cellulase addition on the extraction of cellulose.

The protein was removed by adding proteases, and an ultrasound-assisted enzymatic method was used to analysis the extraction of cellulose. The results showed that the extraction of cellulose was the highest when bean dregs were added with 0.005 g papain (Figure 1A). When bean dregs were added with 0.01 g alkaline protease, the extraction of cellulose could be more than 90% (Figure 1B). When bean dregs were added with 0.05 g trypsin, the extraction of cellulose was the highest (Figure 1C). In addition, there were no significant differences with adding 0.2 g or 0.25 g cellulase for the extraction of cellulose, but 0.2 g cellulase was considered the optimum for practical application considering the costs (Figure 1D). Furthermore, the optimum condition for obtaining cellulose was with 0.005 g papain, 0.01 g alkaline protease, 0.05 g trypsin, and 0.2 g cellulose added. In addition, Zhang et al. (2019) studied the extraction of kidney bean residue fiber by ultrasound-assisted complex enzymes, and the yield of water-insoluble cellulose obtained under the optimal process conditions was 60.11% [11]. Pan et al. studied the extraction of water-insoluble fiber from soybean bean residue by ultrasound-assisted alkali method, and the yield of water-insoluble cellulose was 92.11% under the optimal process conditions [12]. Therefore, compared with previous studies, the cellulose yield of bean residue in our study was equally remarkable. 

### 3.3. Analysis of Water-Holding Capacity from Tofu

**Figure 2:**
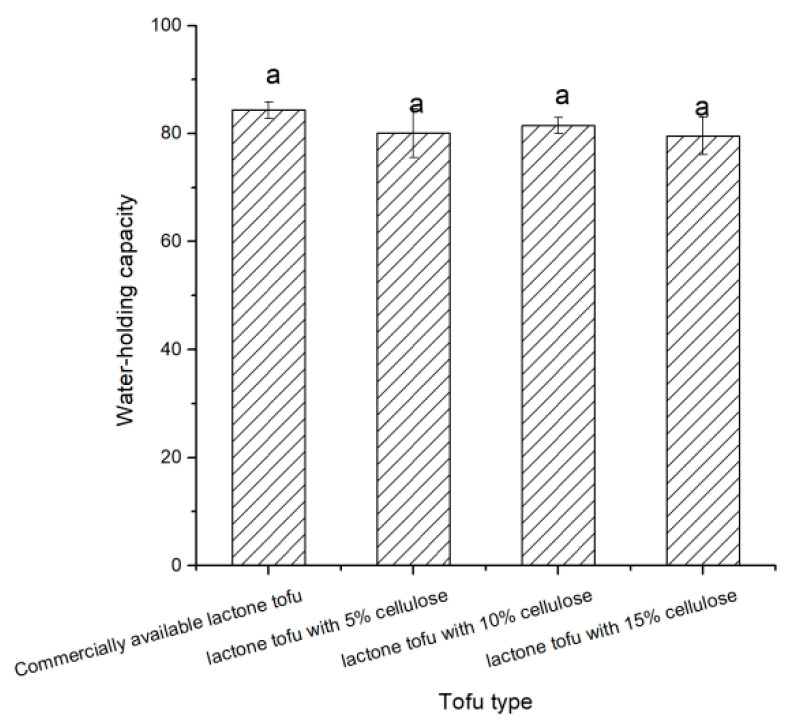
Comparison of the water-holding capacities of the different types of tofu.

Examples of lactone tofu with 5% cellulose, 10% cellulose, and 15% cellulose were produced to compare with the commercially available lactone tofu. Water is an important component of tofu, and it has a very important influence on its quality stability and structural composition [22,24,25]. Water can be coupled to functional groups of polysaccharides and proteins in gel systems, or it can be trapped in microscopic meshes and holes in the gel network [24]. According to Li et al. (2014), tofu’s water-holding capacity is mostly determined by its composition and microstructure [22]. It can be seen in Figure 2 that there were no significant differences in water-holding capacity between the tofu made from the three different processes and the commercially available lactone tofu (*p* > 0.05), showing that the innovative process of making tofu yielded good water-holding capacity. 

### 3.4. Analysis of Magnetic Resonance Imaging

**Figure 3:**
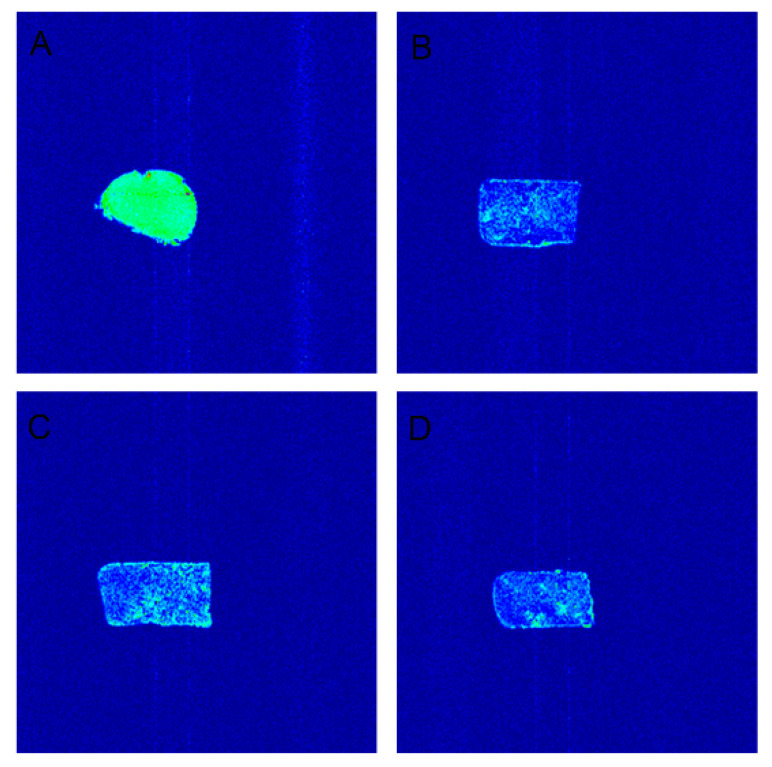
Pseudo color map of different tofu types. (**A**) Commercially available lactone tofu. (**B**) Lactone tofu with 5% cellulose. (**C**) Lactone tofu with 10% cellulose. (**D**) Lactone tofu with 15% cellulose.

The principle of the nuclear magnetic resonance pseudo-color map is to explore the water distribution [22]. It can be seen in Figure 3 that the tofu made from the different processes had the characteristics of uniform distribution. The results showed that whole bean tofu not only had good water retention but also avoided wasting resources and improved the utilization rate and nutritional value of tofu. Under the same process conditions, the pseudo-color map of lactone tofu was brighter, which proved that the moisture content was higher and the water retention was better (Figure 3A). It was clear from Figure 3 that the brightness was higher with 10% addition of soybean residue cellulose than at 5% or 15% addition, proving that the moisture content was greater, while the density of brightness also confirmed that the moisture distribution was tight. Comparing the different soybean residue cellulose additions, lactone tofu with 10% cellulose had the best water retention and highest moisture content (Figure 3C). A previous report indicated that increased solid content seems to harm the moisture content of tofu [25]. Cai et al. (1997) showed that tofu containing a high proportion of solids had low moisture content [26]. A similar result in this study showed that increasing the fiber content of soybean residue to a certain extent can increase the moisture content, but a too-high fiber content can reduce the moisture content of tofu.

### 3.5. Texture Analysis of Tofu Added with Cellulose

**Figure 4:**
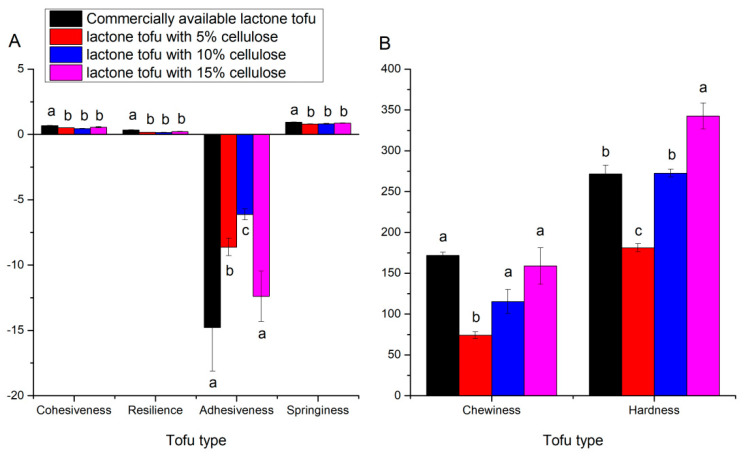
Analysis of full texture of different tofu types. (**A**) represent the texture properties of cohesiveness, resilience, adhesiveness, and springiness. (**B**) represent the texture properties of chewiness and hardness.

The texture indices of tofu are the main characteristics for judging the quality of tofu and are also important conditions for acceptability in the tofu market [27]. Figure 4A and B showed that the commercially available lactone tofu had high cohesiveness, resilience, adhesiveness, and springiness, and there were significant differences in the tofu between the commercially available lactone tofu and the other tofu types (*p* < 0.05). Springiness refers to how well a product physically bounces back after being distorted during the first compression, and products with higher springiness are regarded as having more elasticity [27]. Cohesiveness refers to how well a product can tolerate a second deformation in comparison with the first, which can be interpreted as the tightness of the binding inside the gel to resist the deformation [28,29]. Resilience is an indicator of how a sample recovers from deformation, including both speed and strength [30]. From Figure 4A, it can be seen that the tofu with different amounts of bean fiber added had consistent elasticity that was lower than that of commercially available tofu, and this result also appeared in the characteristics of resilience and cohesiveness (*p* > 0.05). 

During TPA analysis, the absolute peak force on the first downstroke was used to determine hardness [30], and the addition of soybean residue fiber to tofu increased the tofu product’s solid content; adding addition of 10% soybean residue fiber had the same hardness as that of commercial lactone tofu (*p* > 0.05) [31]. Cruz et al. (2009) mentioned that the large fat droplets in soy-yogurt gel disrupt the network homogeneity and decrease the gel’s firmness, whereas adding cellulose can strengthen the hardness of tofu [32]. Moreover, the chewiness of tofu indicates how easy it is to swallow. In this study, tofu with different amounts of bean fiber addition had the same chewiness as commercially available lactone tofu (*p* > 0.05), except for the 5% addition. Adhesiveness is the energy required to break up the attractive forces between the surface of the food and the surfaces of other materials. Figure 4A depicted that only lactone tofu with 15% cellulose had similar adhesiveness to that of the commercially available lactone tofu. Lactone tofu with cellulose contains a large amount of cellulose and soybean polysaccharides, which weakened the interaction between proteins, resulting in a loose network structure of the gel and a reduction in texture indices [30]. In brief, the lactone tofu samples with 10% cellulose and 15% cellulose both had certain market competitiveness in ensuring the quality of tofu.

### 3.6. Chroma Analysis

**Figure 5:**
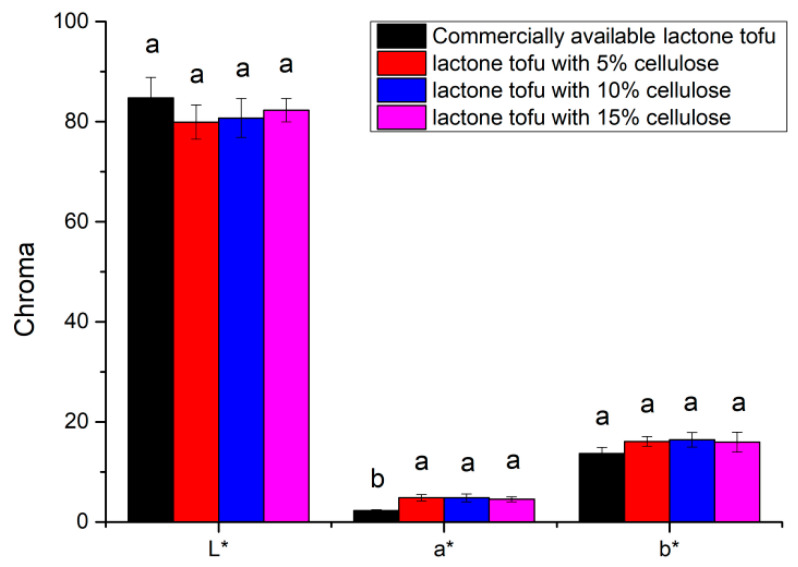
Chroma analysis of the different tofu types.

The best color of tofu is milky white. The larger the value of L*, the brighter the color of the tofu. The larger the value of b*, the yellower the tofu. The larger the value of a*, the much redder the tofu [29]. As shown in Figure 5, the gloss of commercially available lactone tofu was the best, but the color differences between the various tofu was not obvious (*p* > 0.05) except for the a* value of the commercially available lactone tofu. There were no significant differences in the L* and b* values between the various tofus (*p* > 0.05). The color had a certain influence. On the other hand, the composite structure formed by high-speed pulverization of soybeans also affected the refractive index of the obtained tofu [30]. However, except for the commercially available lactone tofu, there were generally no significant differences in the colors of the tofu from the different processes (*p* > 0.05). Murugkar et al. (2014) found that adding fibrous material to tofu darkened it and that increasing the particle size of pulverized sprout seed in soymilk affected the whiteness index of tofu products [33]. However, in this study, there were no significant differences in the lightness between the commercially available and the tofu with different additions of soybean residue fiber.

### 3.7. Microstructure of Tofu

**Figure 6:**
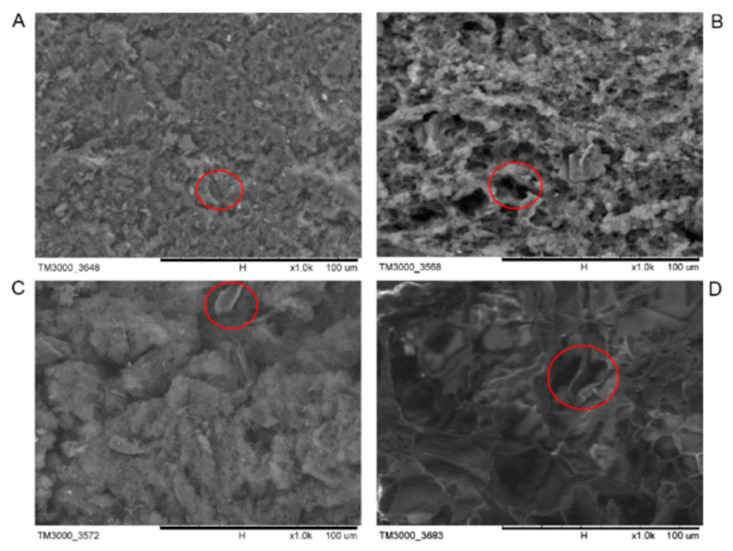
Scanning electron micrographs of tofu made with different additions of soybean fiber and commercially available lactone tofu at 1000× magnification. (**A**) Commercially available lactone tofu. (**B**) Lactone tofu with 5% cellulose. (**C**) Lactone tofu with 10% cellulose. (**D**) Lactone tofu with 15% cellulose. The circles represent cellulose fragments and pores.

The main reason why soybean residue affected the formation of the whole tofu gel network was that the polysaccharide in the soybean residue was adsorbed on the surface of the soybean protein, which decreased the hydrophobic surface area on the protein surface and thus weakened the support of the protein to the gel network [13]. This finally caused the inability of the tofu to form good gel stability (35; Figure 6). However, the tofu from a wet semi-degreasing process generally weakened the effect of the bean dregs on the gel structure and had a good gel structure [34].

For the cellulose-added tofu, as the amount added increased, the hydrophobic effect of the cellulose-attenuated protein was enhanced, resulting in the formation of a fish-scale network structure (Figure 6D) showing a weak gel state. While lactone tofu with 10% cellulose (Figure 6C) presented a better gel network, the bean dreg cellulose had less influence to a certain extent. The result was consistent with findings from Ullah et al. (2019) [17]. The number, distribution, and size of the cavities were affected by particle size and the volumetric ratio of the added soybean dreg fiber [17].

### 3.8. Analysis of Volatile Organic Compound

**Figure 7:**
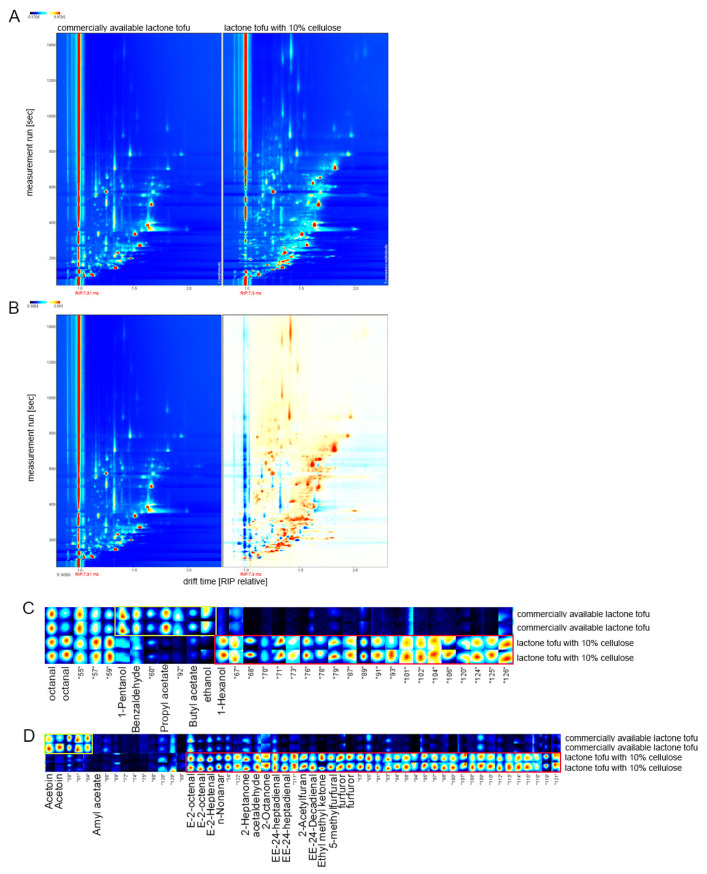
Analysis of the volatile components of the different tofu types. (**A**,**B**) The ordinate represents the retention time of the gas chromatograph, and the abscissa represents the ion migration time. The background of the whole picture is blue, and the red vertical line at 1.0 is the RIP peak (reactive ion peak, normalized). Each point on either side of the RIP peak represents a volatile organic compound. The color represents the concentration of the substance: white indicates a lower concentration, red indicates a higher concentration, and darker color indicates a higher concentration. (**C**,**D**) Each row in the graph represents all signal peaks selected in the tofu sample. Each column in the figure represents the signal peak of the same volatile organic compound in the different tofu samples. The complete volatility information for each sample and the difference in volatile organic compounds between the samples can be seen in the graph.

Combining the results of all the previous experiments, the cellulose addition of 10% was finally selected for judging the aroma differences between the final products and the commercially available lactone tofu. As can be seen from the comparison chart above (Figure 7A,B), the volatile organic compounds in the two kinds of tofu were significantly different. In order to compare the differences between the different samples clearly, a difference comparison mode was employed in the study: The spectrum of commercially available lactone tofu was selected as a reference, and the spectrum of the lactone tofu with 10% cellulose was deducted from the reference. If the volatile organic compounds were the same, the deducted background was white, while red indicated that the concentration of the substance was higher than the reference, and blue indicated that the concentration of the substance was lower than the reference [18].

The content of the above-mentioned fingerprint red box for the lactone tofu with 10% cellulose was much higher than the commercially available lactone tofu, including 1-pentanor, benzaldehyde, prophy acetate, butyl acetate, ethanol, and acetoin (Figure 7C). The substance in the yellow box was much more present in the commercially available lactone tofu, comprising 1-hexanor, E-2-octenal, E-2-heptenal, n-nonanar, 2-heptanone, phenylacetaldehyde, 2-octanone, EE-24-heptadienal, 2-acetylfuran, EE-24-decadienal, ethyl methyl ketone, 5-methylfurfural, and furfuror (Figure 7D), which contributed strong flavor to the commercially available lactone tofu [20]. These results suggested that a fingerprint of the flavor components of high-fiber tofu can be successfully established using GC-IMS, and this result was confirmed in a related study by Yang et al. (2021) [18].

## 4. Conclusions

In order to take full advantage of bean dregs to improve the quality of tofu, ultrasound-assisted protease was used to prepare soybean residue cellulose. The results showed that there was no statistically significant difference between commercially available lactone tofu and high-fiber tofu in water-holding capacity, texture characteristic, or color. Additionally, this high-fiber tofu was superior in aroma composition but inferior in moisture content and gel structure. Comprehensively considering the above, it was suggested that lactone tofu with 10% cellulose would have the best market competitiveness. In all, the results of this paper provided a theoretical basis for future commercial developments in improving tofu quality.

## Figures and Tables

**Table 1:** The content of nutrients in bean dregs.

Nutrients (g/100 g)	Content
Moisture	84.8 ± 1.03
Ash	0.38 ± 0.19
Protein	2.18 ± 0.89
Fat	0.3 ± 0.01
Crude fiber	3.8 ± 0.91

## Data Availability

Not applicable.
